# Trigeminal Neuralgia Triggering Recurrent Attacks of Migraine in a Chronic Migraineur: A Hidden Etiology Between the Lines

**DOI:** 10.7759/cureus.55028

**Published:** 2024-02-27

**Authors:** Hussein Harb, Pranav Chaudhari, Sourya Acharya, Sunil Kumar, Rucha Sawant

**Affiliations:** 1 Department of Elective Rotations, Ross University School of Medicine, Bridgetown, BRB; 2 Department of Internal Medicine, Jawaharlal Nehru Medical College, Datta Meghe Institute Of Medical Sciences, Wardha, IND; 3 Department of Medicine, Jawaharlal Nehru Medical College, Datta Meghe Institute of Medical Sciences, Wardha, IND; 4 Department of Internal Medicine, Jawaharlal Nehru Medical College, Datta Meghe Institute of Medical Sciences, Wardha, IND

**Keywords:** initial diagnosis, diagnostic errors, anticonvulsants, facial pain, migraine, trigeminal neuralgia

## Abstract

A 25-year-old man with a medical record of migraines presented with progressively worsening episodes of dizziness and constant right-sided neck and facial pain, each lasting for hours over a month. The increased pain frequency and lack of response to abortive treatment were not typical of his migraines. Investigation for an alternate cause of the patient's symptoms with an MRI revealed evidence of trigeminal neuralgia (TN). Following TN treatment and complete resolution of symptoms, the diagnosis was confirmed. The atypical presentation of TN and the existence of migraine history and symptoms in this patient suggest a relationship between TN episodes triggering migraine. This case underscores the importance of considering atypical presentations in patients with a history of migraines and the potential interplay between TN and migraines in diagnosis and treatment.

## Introduction

Trigeminal neuralgia (TN) is a consequence of irritation of the trigeminal nerve, which is responsible for unilateral facial sensation. It is a rare pathology that presents as a facial pain disorder along the trigeminal nerve distribution, affecting the jaw, nose, and area above the eye. This results in the typical clinical signs of sporadic, paroxysmal stabbing pain lasting from a second to two minutes. Pain episodes may be spontaneous or precipitated by triggers such as face washing, exposure to a cold breeze, or speaking [1,2]. Per the International Classification of Headache Disorders, 3rd edition (ICHD-3), TN may be diagnosed if these signs and symptoms are present and cannot be better explained by an alternative diagnosis [3]. Conversely, migraine is a significant global disability affecting over a billion people. The diagnostic criteria for migraine require at least five headache episodes lasting between four and 72 hours. These episodes must exhibit at least two of the following characteristics: a unilateral location, pulsatile, moderate to severe intensity, and exacerbated by routine physical activity [3]. Additionally, each headache episode should include at least one of the following symptoms: nausea and vomiting, or photophobia and phonophobia. These symptoms should not be more accurately attributed to another diagnosis [3-5]. Vestibular migraine is diagnosed when moderate-to-severe vestibular symptoms (e.g., vertigo and nystagmus) accompany migraine episodes for five minutes to 72 hours [3]. This case report is about a 25-year-old man with a medical history of migraine. Upon history further analysis, it was discovered that his symptoms did not completely correspond with the common presentations associated with either TN or migraines, drawing attention to the intricate nature of diagnosing these conditions when they overlap. The intersection of these two conditions, in this case, presents a unique opportunity to explore the diagnostic and management complexities when typical presentations are absent.

## Case presentation

The patient, a 25-year-old man with a medical history of migraine, arrived at the Casualty Department with a one-month account of insidious onset, progressively worsening vertigo and right-sided hemicranial pain. The patient described intermittent vertigo, not linked to syncope, neck movements, or postural changes, but exacerbated by eight episodes of hemicranial facial pain. The pain originated from the right posterior mastoid, radiated to the parietal and temporal regions, then to the lower jaw, and occasionally to the forehead. It was described as throbbing and constant, with episodes of increased severity lasting four hours. These episodes of pain were accompanied by occasional, irregular, electric-like stabbing pains along the mandible, lasting for seconds. The generalized pain worsened while chewing hard foods and riding a motorcycle with wind exposure to the face. The pain subsided briefly for a few days following a self-initiated trial of two to three tablets of 400 mg ibuprofen tablets daily. However, symptoms then recurred 10 days ago, accompanied by additional symptoms of phonophobia. 

The patient's medical record is significant for three years of migraine diagnosed by a neurologist. The patient experiences three to four episodes of migraine attacks every six months. These attacks are typically unilateral and pulsatile; however, they are less severe, with reduced vertigo, and are associated with both photophobia and phonophobia. Migraines were controlled with prophylactic propranolol of 40 mg daily and SOS sumatriptan therapy. Surgical history is significant only for a dental procedure three months prior. There were no known allergies, medications, significant family history, or tobacco/alcohol use. A review of systems was negative for trauma, loss of consciousness, seizures, chest pain, gastrointestinal discomfort, altered appetite, tinnitus, or earache. The patient’s vitals were within normal limits, except for elevated blood pressure of 155/95 mmHg. Neurological examinations revealed no cranial nerve deficits, no motor deficits, and intact sensation bilaterally. ECG findings were within acceptable limits. 

**Figure 1:**
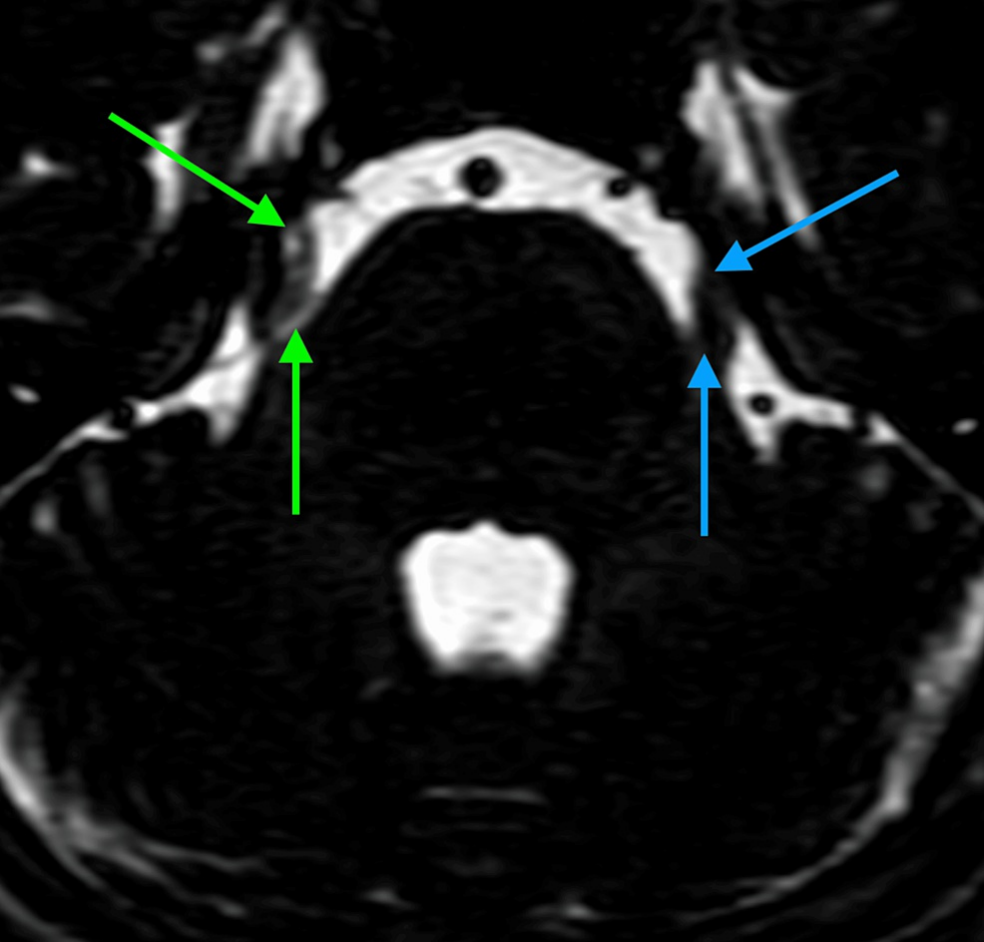
Bilateral comparison of the trigeminal nerve with FIESTA protocol in the axial section of the brain at the level of pons. Green arrow: right branch of the superior cerebellar artery abutting the right trigeminal nerve. Blue arrow: unaffected trigeminal nerve. FIESTA, fast imaging employing steady-state acquisition

The diagnosis of migraine was confirmed based on international guidelines [3]. Migraine treatment was started with 100 mg of sumatriptan abortive treatment. However, after two doses over two days and no improvement in symptoms, we suspected an alternate cause. Brain MRI revealed the right branch of the superior cerebellar artery compressing the transitional zone of the right trigeminal nerve (Figure 1). Given the pathognomonic finding indicating TN, we proceeded with the first-line treatment of oxcarbazepine of 150 mg orally twice daily. The patient was also started on gabapentin 300 mg once daily as prophylaxis for migraine [6]. After two days of treatment, the patient's symptoms had almost completely resolved, and the diagnosis of TN was confirmed. Neurosurgery consults recommended continuation of medical therapy, with no surgery advised given appropriate symptom control. Given the patient's clinical timeline, we suspected that TN pain episodes were triggering migraines. The patient was discharged without any complaints or adverse effects from the medications. The prescribed dosages of oxcarbazepine for TN and propranolol for migraine prophylaxis were continued, as mentioned above. At one month follow-up, the patient reported one episode of facial, hemicranial pain of decreased severity relative to previous attacks.

## Discussion

TN, a condition profoundly affecting the quality of life, manifests in two primary forms: classical trigeminal neuralgia (CTN) and secondary trigeminal neuralgia (STN). CTN, commonly referred to as tic douloureux, is the prevalent form, with an incidence rate of about 4.5 out of 100,000 individuals per year [1,2]. In contrast, STN constitutes 14%-20% of TN cases and is typically associated with conditions like multiple sclerosis or structural abnormalities such as tumors or aneurysms [7,8].

The pathophysiology of TN is often rooted in ultrastructural anomalies in the nerve root area, especially where vascular compression is evident. These anomalies include the focal loss of myelin, juxtaposed demyelinated axons, a decrease in oligodendrocytes, and an absence of inflammatory cells. Although neurovascular conflict leading to demyelination is a significant contributor in many CTN cases, the exact etiology remains partially unknown, as only about half of CTN patients exhibit such morphological changes [9]. Other rare causes such as meningioma manifest as STN via mass effects [10].

Clinically, TN is characterized by brief, recurrent, and paroxysmal attacks of severe pain, resembling an electric shock. The pain, which is sharp, shooting, or stabbing in nature, is often triggered by minor stimuli and affects one side of the face [7].

Conversely, migraine episodes last from four to 72 hours and are usually one-sided, pulsatile headaches along with vomiting, photophobia, and phonophobia. Notably, a majority of migraines occur without aura, although a subset of patients experience a prodromal sensory or visual disturbance [11]. The pathophysiology involves cerebral vascular changes and the release of vasoactive neuropeptides, influenced by a variety of environmental and physiological factors [12]. Propranolol stands as a first-line prophylactic treatment, while acute interventions include analgesics, nonsteroidal anti-inflammatory drugs (NSAIDs), ergot derivatives, and triptans [3,13].

In this case, the patient's complex symptomatology, including progressive vertigo, persistent unilateral pain, and previous migraine history, initially suggested vestibular migraine headaches. However, the emergence of additional symptoms like brief, occasional, irregular, electric-like stabbing pains along the mandible facial pain and exacerbation by external stimuli necessitated a comprehensive diagnostic approach.

MRI imaging plays a pivotal role in the TN diagnostic process, distinguishing between symptomatic and classical forms and guiding subsequent management. It's imperative as clinical assessment alone may not provide a definitive diagnosis, and symptomatic TN warrants targeted treatments. Differential diagnoses encompass various facial pain syndromes, highlighting the need for precise and thorough evaluation [14].

The patient's clinical evolution, marked by a decrease in migraine episodes following targeted TN treatment, suggests a correlation between TN episodes and subsequent migraine attacks. This case underscores the importance of addressing potential underlying conditions like TN in patients with complex headache disorders.

TN management begins with the assessment of renal, liver, and sodium levels. First-line management typically involves sodium channel-inhibiting medications like carbamazepine or oxcarbazepine, known for their efficacy [15-17]. While carbamazepine is effective, it is associated with side adverse effects like somnolence, dizziness, and rash. Oxcarbazepine, on the other hand, is often favored over carbamazepine because it generally has a better side effect profile and fewer drug interactions, making it more tolerable for patients [18]. Should these treatments prove ineffective or intolerable, alternatives like gabapentin, lamotrigine, or baclofen are recommended. Persisting treatment challenges may necessitate surgical consultation, as advised by international guidelines [19]. Options for surgical management include rhizotomy, microvascular decompression, and stereotactic radiosurgery [20]. Surgical option selection is tailored to individual circumstances, including patient preferences, adverse risk-to-benefit ratio, and available expertise. 

## Conclusions

The presented case illustrates the possible relationship between TN and migraines and the challenges of making a diagnosis of pathologies with similar presentations. Moreover, it shows the importance of a comprehensive evaluation with consideration of TN in patients with atypical headache disorders. Finally, this case demonstrates the success of TN treatment and its potential effectiveness in comorbid conditions such as migraine.

## References

[1] Araya EI, Claudino RF, Piovesan EJ, Chichorro JG (2020). Trigeminal neuralgia: basic and clinical aspects. Curr Neuropharmacol.

[2] Katusic S, Beard CM, Bergstralh E, Kurland LT (1990). Incidence and clinical features of trigeminal neuralgia, Rochester, Minnesota, 1945-1984. Ann Neurol.

[3] (2018). Headache Classification Committee of the International Headache Society (IHS) The International Classification of Headache Disorders, 3rd edition. Cephalalgia.

[4] Ornello R, Andreou AP, De Matteis E, Jürgens TP, Minen MT, Sacco S (2024). Resistant and refractory migraine: clinical presentation, pathophysiology, and management. EBioMedicine.

[5] Giri A, Acharya S, Kumar S (2021). Study of clinical profile and association of migraine with dyslipidemia. J Pharm Res Int.

[6] Pringsheim T, Davenport W, Mackie G (2012). Canadian Headache Society guideline for migraine prophylaxis. Can J Neurol Sci.

[7] Maarbjerg S, Gozalov A, Olesen J, Bendtsen L (2014). Trigeminal neuralgia--a prospective systematic study of clinical characteristics in 158 patients. Headache.

[8] Cruccu G, Biasiotta A, Galeotti F, Iannetti GD, Truini A, Gronseth G (2006). Diagnostic accuracy of trigeminal reflex testing in trigeminal neuralgia. Neurology.

[9] Hilton DA, Love S, Gradidge T, Coakham HB (1994). Pathological findings associated with trigeminal neuralgia caused by vascular compression. Neurosurgery.

[10] Izzati-Zade KF (2008). The role of serotonin in the pathogenesis and clinical presentations of migraine attacks. Neurosci Behav Physiol.

[11] Sesha Satya Sagar VV, Acharya S, Andhale A, Kumar S, Talwar D (2022). A case of suprasellar, intrasellar, and Infrasellar meningioma presenting as a visual field defect in an obese female. Cureus.

[12] Skaer TL (1996). Clinical presentation and treatment of migraine. Clin Ther.

[13] Kumar A, Kadian R (2024). Migraine Prophylaxis.

[14] Cruccu G, Gronseth G, Alksne J (2008). AAN-EFNS guidelines on trigeminal neuralgia management. Eur J Neurol.

[15] Wiffen PJ, Derry S, Moore RA, McQuay HJ (2011). Carbamazepine for acute and chronic pain in adults. Cochrane Database Syst Rev.

[16] Killian JM, Fromm GH (1968). Carbamazepine in the treatment of neuralgia. Use of side effects. Arch Neurol.

[17] Nicol CF (1969). A four year double-blind study of tegretol in facial pain. Headache.

[18] Schmidt D, Elger CE (2004). What is the evidence that oxcarbazepine and carbamazepine are distinctly different antiepileptic drugs?. Epilepsy Behav.

[19] Nurmikko TJ, Eldridge PR (2001). Trigeminal neuralgia--pathophysiology, diagnosis and current treatment. Br J Anaesth.

[20] Cruccu G, Di Stefano G, Truini A (2020). Trigeminal neuralgia. N Engl J Med.

